# Translational considerations in injectable cell-based therapeutics for neurological applications: concepts, progress and challenges

**DOI:** 10.1038/s41536-017-0028-x

**Published:** 2017-08-10

**Authors:** Mahetab H. Amer, Felicity R. A. J. Rose, Kevin M. Shakesheff, Michel Modo, Lisa J. White

**Affiliations:** 1 School of Pharmacy, University of Nottingham, Nottingham, NG7 2RD UK; 2 McGowan Institute for Regenerative Medicine, University of Pittsburgh, Pittsburgh, PA USA; 3 Department of Radiology, University of Pittsburgh, Pittsburgh, PA USA

## Abstract

Significant progress has been made during the past decade towards the clinical adoption of cell-based therapeutics. However, existing cell-delivery approaches have shown limited success, with numerous studies showing fewer than 5% of injected cells persisting at the site of injection within days of transplantation. Although consideration is being increasingly given to clinical trial design, little emphasis has been given to tools and protocols used to administer cells. The different behaviours of various cell types, dosing accuracy, precise delivery, and cell retention and viability post-injection are some of the obstacles facing clinical translation. For efficient injectable cell transplantation, accurate characterisation of cellular health post-injection and the development of standardised administration protocols are required. This review provides an overview of the challenges facing effective delivery of cell therapies, examines key studies that have been carried out to investigate injectable cell delivery, and outlines opportunities for translating these findings into more effective cell-therapy interventions.

## Introduction

Significant progress has been made during the past decade towards the clinical adoption of cell-based therapeutics. Pre-clinical studies have translated into clinical trials for conditions of the central nervous system (CNS), including Parkinson’s disease (PD),^1^ Huntington’s disease,^2^ amyotrophic lateral sclerosis (ALS)^3^ and stroke.^4, 5^ Clinical trials have focused on the delivery of purified cellular suspensions, for example, in spinal cord injuries and stroke.^6–8^ However, existing cell-delivery approaches have shown limited success, with numerous studies showing fewer than 5% of injected cells persisting at the site of injection within days of transplantation.

One of the main translational challenges to the implementation of injection-based cell therapy is the need to determine suitable delivery protocols to ensure sufficient accuracy, improved cell survival and reproducibility in administering cells for therapeutic efficacy.^9^ In this review, we identify critical considerations for the various stages of cell administration, outline studies that have measured functional performance of injected cells and discuss criteria for designing cell-delivery devices for minimally invasive cell therapy. The various approaches used to attempt to maximise cell viability and functionality in high accuracy cell-therapy applications are also described. We suggest that if the variables linked to optimal cell, survival can be recognised, cell loss may be reduced and efficacy of cellular therapies can be improved.

## Cells as therapeutic agents: translational barriers in neurological applications

**Fig. 1:**
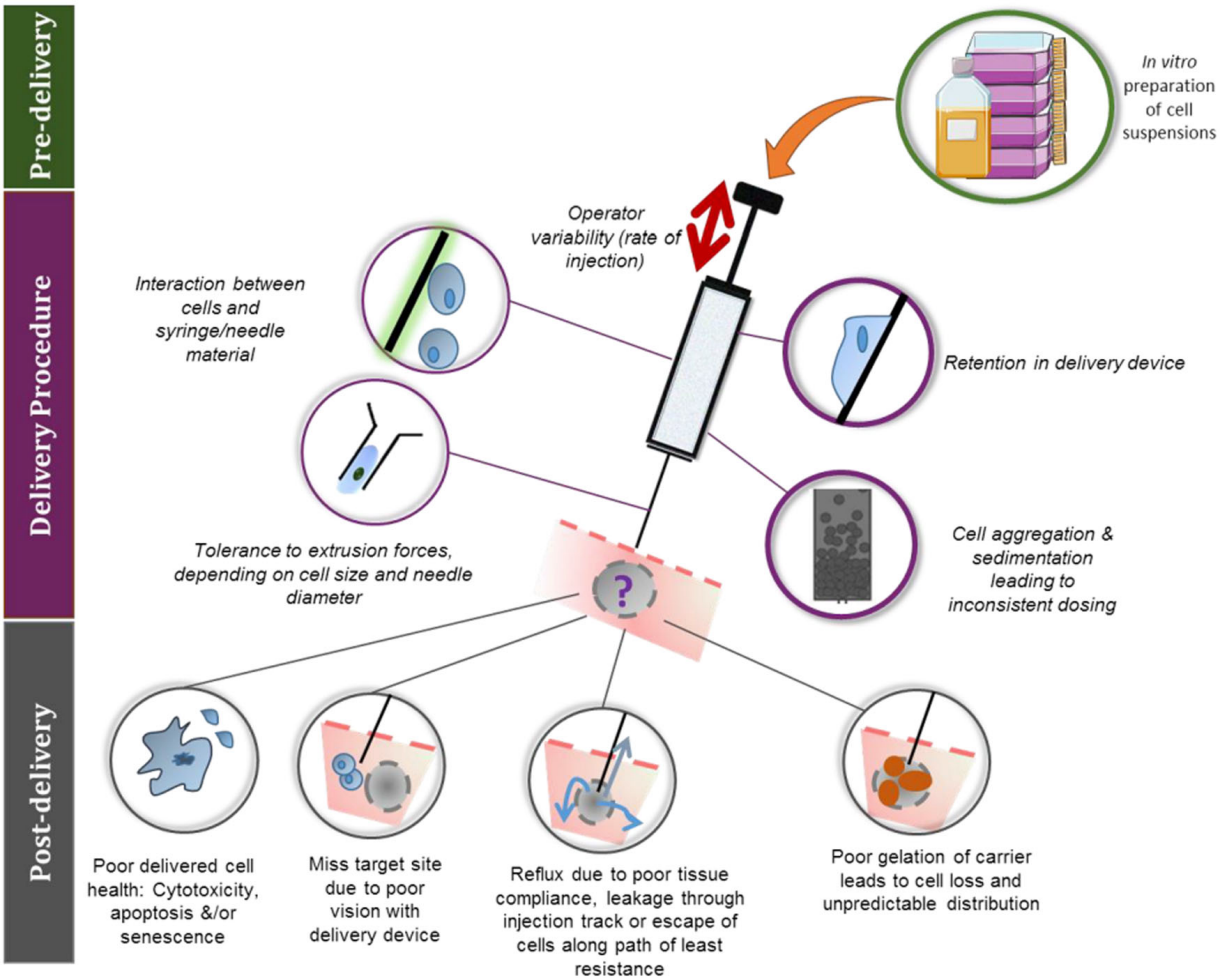
Common problems with injectable cell delivery and possible cell fates. Three stages make up a typical cell-therapy protocol: in vitro preparation (pre-delivery), injection (delivery) and subsequent retention (post-delivery) of injected cells

Three stages make up a typical cell-therapy procedure: (1) in vitro preparation of cell suspensions; (2) injection procedure; and (3) retention of the administered cells post-injection.^10^ Focusing on one stage only can yield optimised settings that are not favourable to the entire procedure, and therefore it is essential that a systematic investigation considers all three stages to outline optimal transplantation parameters (Fig. 1).

Cell loss has been reported to be observed post-transplantation,^11, 12^ with quantified survival rate of transplanted cells as low as 1%.^13^ Moreover, a large number of cells that have been originally retained die, possibly due to exposure of cells to the inflammatory microenvironment, washout, immune destruction, dispersion through impaired local vascular system,^14, 15^ apoptosis and anoikic cell death.^16^ Variable clinical outcomes observed in two trials for PD^1, 17^ have been partially ascribed to a failure to properly distribute cells to the target site.^18^ Attaining efficient delivery of an adequate number of cells without loss of functionality is therefore a key step in the development of regenerative medicine approaches.

The diverse behaviours of various cell types, choice of dosing density, administration protocol and cell viability post-injection are some of the obstacles facing clinical translation. This section will explore the various variables involved in the three stages of cell-therapy procedures.

### Pre-delivery factors: scaling up pre-clinical models to human therapy

To overcome low cell transplantation efficiency, one popular approach to translational scale-up has been to deliver a large number of cells to a single site^19^ with doses ranging up to hundreds of millions of cells.^19^ This makes cell-therapy approaches technically complex and expensive, as well as offering limited control over site-specificity, as cells will potentially migrate to other sites.^20^

*Determination of cell density*. In cell transplantation studies, cell concentration within suspensions is often reported, with suspensions of over 100,000 cells/μL considered highly concentrated.^21^ As well as higher costs of cell processing, these suspensions can be viscous and may cause needle clogging and uneven injection flow. Moreover, as cell size varies widely, depending on the site or species of origin, the maximum concentration suspended within a certain volume will therefore vary. Hence, it is more accurate to express the cellular component as a volume fraction, which is the volume occupied by the cell fraction in a certain volume of suspension vehicle, as described by Rossetti et al.^10^

High-density cell suspensions may lead to cell death, attributable to limited oxygen and nutrient diffusion.^22^ The limited capacity to generate appropriately large numbers of standardised cells routinely, within a limited time frame, whereas adhering to quality standards has also led to small clinical trials. In addition, large doses of administered cells also pose higher safety risks of tumorigenesis^23^ and micro-embolism.^24^ Moreover, higher injected cell concentrations result in exposure to increased shear forces,^25^ yet may have a reduced tendency for sedimentation.^10^

No agreement exists regarding the optimal cell number to be transplanted, although this is likely to vary depending on cell type, disease and administration route. For example, 3–5 × 10^7^ cells/kg mesenchymal stem cells (MSCs) were administered per multiple sclerosis patient,^26^ whereas in spinal cord injury, 5–6 × 10^6^ cells/kg have been transplanted intrathecally.^27^ Taguchi et al.^28^ reported that the higher dose of 3.4 × 10^8^ cells gave improved neurologic outcomes than the lower dose of 2.5 × 10^8^ cells administered intravenously. A lower percentage loss of MSCs was exhibited with increasing concentrations being administered, possibly due to the finite number of cells being able to attach to the available inner surface of the device.^29^ However, a phase II trial using 5–10 million NT2N cells in stroke patients showed that patients receiving the lower dose performed better.^4^ A recent study also demonstrated that a suitable cell dose, rather than a higher one, can better aid the repair of injured tissue in stroke patients.^30^

*Cell suspension vehicle*. Suspension vehicles have been found to affect the viability of cells pre-delivery and their survival upon implantation.^10, 31, 32^ Preparing a cell suspension that maintains a homogeneous distribution and viability is essential to ensure effective clinical translation. Results suggested that MSCs viability is reduced to levels significantly below the permitted limit of 70% in a short time when stored in parenteral solutions, with other biological functions being slightly affected.^31^ Cells at different temperatures will also have different requirements for storage solutions.^33^

*Injection volume*. The human brain is 800–2300 times larger than that of rodents used for pre-clinical research.^34^ To enable scale-up to larger target volumes, cell distribution can be increased by making multiple, lower volume injections for improved engraftment.^14^ The adjustment of the needle/catheter for adequate cell distribution can lead to multiple needle tracks and entry points. Multiple transcortical brain penetrations have been used for a range of clinical trials.^1, 17, 35^ This is a cause for concern, as each penetration carries a risk of intracranial haemorrhage^22^ and damage to white matter tracts.

The shortage of human studies with lesion volume calculations, such as occur in human spinal cord injuries,^36^ make it challenging to decide on optimal injection volumes. A study by Gutierrez et al.^37^ evaluated the spinal cord’s tolerance to varying numbers and volumes of cell injections in Göttingen minipigs. Complete functional recovery was achieved by 2 weeks, even when injection volume and numbers were increased. However, histological analysis revealed tissue damage when large volumes (50 µL) of cell suspension were injected per site. Although increased numbers of injections did not cause an increase in tissue damage there was an optimal number of injections required to achieve the best engraftment.

In many clinical trials, doses are extrapolated from data of pre-clinical animal studies. Depending on how they are calculated, doses of 100 million or more cells may result in substantial volumes being required. Thus, it is vital that detailed dosing studies are carried out in animal models to establish the minimum effective and maximum tolerated doses.^38^

### Cell-delivery challenges

*Cell injector system design requirements and challenges*. The main delivery platform for cell-based therapeutics has traditionally been a needle and syringe,^39^ with cell preparations delivered either systemically or directly.^40^ Although many clinical trials have used syringe/needle systems without cannulas,^41–44^ CNS cell transplantation trials have typically utilised a frame-based platform for the insertion of a stereotactically guided straight cell-delivery cannula or needle.^1, 2, 17, 22, 35, 44–46^ There is a growing recognition that conventional needle-based and catheter-based cell transplantation tools have considerable inadequacies that may affect clinical translation.^47, 48^ Insufficient pre-clinical testing of surgical tools and methods for cell delivery to the human brain and spinal cord may result in the failure of cell transplantation trials, despite the reliability of the basic biological concepts.^49–51^ Key considerations for clinical translation of cell-delivery devices include ease of loading and use, reproducibility of delivery, possibility of sterilisation, freedom from leachable and/or extractable contaminants, and ensuring no visual obstruction through a surgical microscope in high accuracy applications.

*Role of mechanical forces* The mechanical forces that cells experience as they pass through the injection device is a factor influencing their subsequent viability and functionality post-transplantation. To comprehend the fluid dynamics in action, we must explore the mechanical forces exerted on the cells.

**Fig. 2:**
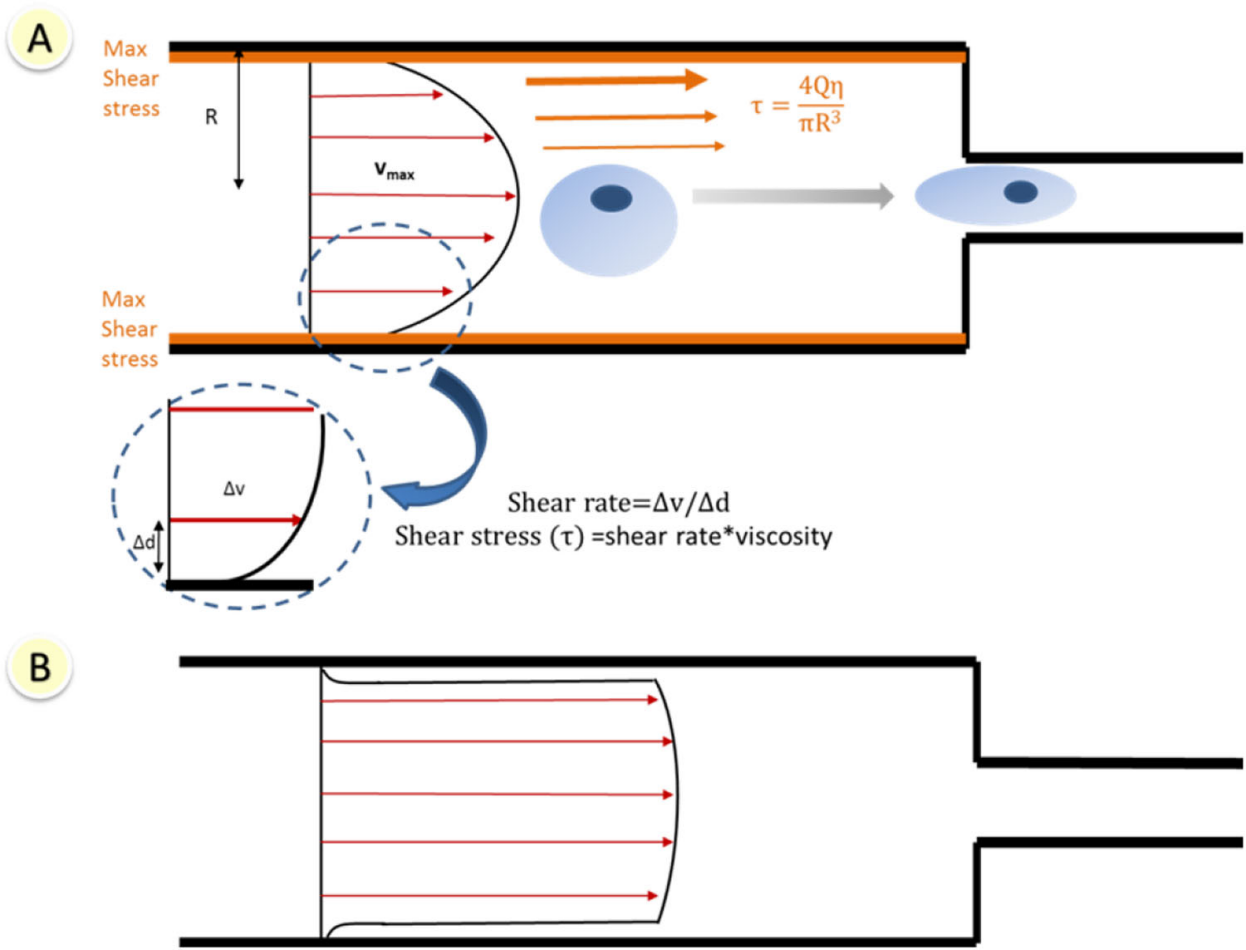
Schematic of a section of a syringe/needle lumen of radius *R*. **a** Shear stress and velocity distribution in delivery device for Newtonian fluid and laminar flow. The velocity profile across the diameter is parabolic. Shear stress (*τ*) is zero at the centre and increases linearly to its maximum value at the wall. As a cell flows from the syringe to the needle, it will experience increasing velocities along its length, causing it to stretch. **b** Plug flow behaviour—flow velocities are almost equal across the whole diameter. Shear-thinning materials display this behaviour when their flow in a capillary reaches a steady state

While flowing through a needle, cells may experience several types of mechanical forces, comprising shear forces characteristic of linear shear flow, a pressure drop across the cell and extensional (stretching) forces.^52^ The nature of flow, whether laminar or turbulent, should be confirmed at the ejection rate and syringe/needle size used for the transplantation procedure. This can be verified through the calculation of Reynold’s number (*R*_e_), which determines flow conditions (transitional level to turbulence is *R*_e_ = 2100):

$${R_{\rm{e}}} = \frac{{\rho Q}}{{15\pi D\eta }},$$

where *ρ* is the carrier fluid density (water at room temperature=999.97 kg/m^3^), *Q* is volumetric flow rate (mL/min), *D* is needle diameter and *η* is dynamic viscosity of the medium. Given that the flow is laminar, the velocity profile is parabolic across the diameter (Fig. 2a), with maximum velocity at the centre of the lumen. Cells and fluid in the middle of a cannula flow at a different velocity to those at the walls. This difference in velocity exposes cells to shear stress.^25^ Changes in shear rate and shear stress have been suggested to affect cell viability and function.^52^ Shear stress (*τ*) is calculated by Poiseuille’s equation,

$$\tau = \frac{{4Q\eta }}{{\pi {R^3}}},$$

where *τ*_max_ is shear stress (dyn/cm^2^); *Q* is flow rate (cm^3^/s); *η* is dynamic viscosity of the medium; and *R* denotes needle radius. The magnitude of shear stress is maximal at the walls of the syringe/needle, zero at the centre and changes linearly with distance between those two. Even low levels of shear stress (10 dynes/cm^2^) have been stated to have a major influence on the activation of molecular cascades.^53, 54^ Any change in the system’s geometry, such as the sudden tapering of a syringe to the needle, can also result in cells experiencing extensional flow, an increase in velocity and, consequently, high shear.^55^ The range of shear stress values generated by clinicians may exceed physiological values. As a useful reference, average wall shear stress is 1–6 dyn/cm^2^ for venous circulation and 15 dyn/cm^2^ for arterial circulation.^56, 57^ However, previous reports have also stated that low shear forces of 3.5 ^58^ and 15 dyn/cm^2^ ^59^ can influence cells. Previous work has concluded that cell damage is based on the extent of shear stress as well as exposure time to zones of shear.^25, 60^ Damage may also occur due to collisions with the stationary surfaces of the device.^61^ Complete damage of the cell may not necessarily be the only adverse result. Investigations carried out on erythrocytes have shown that excessive stretching or deformation of the cell membrane might result in loss of function.^62^ Extensional flow also causes cells to experience stretching and deformation, leading to cell death.^63, 64^ A larger difference between diameters of the syringe and needle will result in larger extensional forces, whereas a longer needle will increase the time a cell is exposed to extensional forces. In addition, cell aggregation may intensify shear stress experienced by cells during delivery. Forces acting on cells during their administration may have two effects: cell destruction along with stretch pre-conditioning.^65^

Biomaterial-assisted cell delivery (reviewed in detail in ‘Biomaterial-assisted delivery’ section) has the potential to mediate the impact of mechanical forces during cell delivery. Shear-thinning hydrogels are injectable materials that exhibit a viscosity decrease under shear strain; the viscosity returns to normal when the shear is removed. Shear-thinning materials typically display plug flow behaviour when their flow within a capillary reaches a steady state. They exhibit a central wide region, where material and cells experience little or no shear rate and a narrow zone of shear adjacent to the walls (Fig. 2b).

*Needle characteristics* Critical parameters in injectable cell delivery include needle characteristics and/or diameter of the tubing used.^50^ Needle characteristics include inner and outer diameters, length, stiffness and bevel design. Deep subcortical target structures, such as the caudate nucleus or corpus striatum, require a long, thin needle/cannula of sufficient rigidity to penetrate to the target site without injuring the overlying structure. This cannula can be 19 cm or more. Shorter needles (8–10 cm) would require direct brain exposure, which is more invasive.^50^

Various needle gauge sizes have been used for neurological applications, typically starting from 25 G.^45, 66^ Devices with needle sizes of 30 G have been developed for CNS applications,^51, 67^ and 30–34 G are typically used for applications requiring high accuracy.^68, 69^ However, smaller bore size needles, e.g.: 27–34 G, are more prone to obstruction by concentrated cell suspensions, especially when successive injections are required. Smaller gauge needles cause less tissue trauma, minimise leakage through the track created by needle insertion, and have been suggested to reduce gliosis.^70^ Mehta et al.^71^ reported a 53% headache rate with the use of a 22 G needle in neurological patients, and suggested that this high incidence could be caused by the large-bore spinal needle, which was used to prevent mechanical damage to the infused cells. Karussis et al.^72^ also reported a high incidence of headache with MSCs in multiple sclerosis and ALS patients, attributing this to the lumbar puncture itself; needle size was not reported. Care should be taken to choose the correct needle size that accounts for cell type, injection speed, site of transplantation and the viscosity of the suspension. Veraitch et al.^73^ reported that 45 passages through a bore of 500 µm diameter were needed before a significant reduction in viability was detected. Longer, finer needles have resulted in the delivery of a smaller volume^50^ and a lower percentage of the cell dose administered.^60^ However, loss of cell viability resulting from the use of smaller needle gauges was not substantial.^25, 60, 74^ Faster acceleration of fluid and cells within longer needles may be more prone to clogging, perhaps because there is less time for cells to dislodge from transient adhesions to other cells and the injector.^50^ Bevel angle and length may also influence injectate dispersion and direction.

*Material employed for construction of delivery device* Recent studies have shown that cells may be retained in the delivery device,^60, 75^ with this effect being more pronounced in glass cannulas than in metal.^75^ Whether the adherence of the cells to the walls of the syringe/cannula is due to chemical, physical or charge effects has not been studied, but all of these might possibly be manipulated to decrease adhesion and settling.^75^ In addition, coating the inner surface of the needle/catheter with proteins may reduce cell adhesion to the device.^76^ In addition to possible adherence to device materials, cells may encounter residual trace amounts of manufacturing agents that could induce apoptosis or undesired differentiation. The use of hydrophilic and siliconised coatings on internal walls of the cannula has also been suggested to decrease friction between the needle and tissue in vivo.^75^ Although glass cannulas present several advantages, including higher precision and minimal penetration injuries,^21^ glass may not be sufficiently rigid to endure injection pressure. Novel materials such as electroactive polymers and magnetorheological elastomers^77^ show promise for the development of tailored needles whose rigidity could be adjusted by current, magnetic field or temperature.

*Sedimentation* Uniformity of cell dosing may be affected by aggregation or sedimentation of cells in suspension over time, with clumping of cells being found to affect cell viability.^78^ The possibility of cell sedimentation during the course of the surgical procedure, especially in large diameter needles/cannula,^75^ and the potential for inconsistent cell dosing needs to be considered in designing cell-therapy protocols, particularly given that orientation will vary depending on application. In vertical cannulas, cells sediment towards the tip of the needle, with most cells appearing in the first 10–20% of the injectate. In horizontal cannulas, settling and adherence of cells on the side caused most of the cell dose to emerge in the final 10–20% of injectate^75^; similar effects of cell dose retention have been reported.^74^ A suspension medium should preferably have a density of ~2 g/cm^3^ and a viscosity >3 cP to reduce cell settling at 20–40% cell volume fraction.^10^ Negative impacts of cell sedimentation observed post cell therapy include graft-induced dyskinesias in PD trials which may have occurred due to formation of dopamine ‘hot spots’ in the brain, resulting in abnormal activation of neural circuits.^79^ Moreover, some cells, such as neural progenitor cells (NPCs), have a natural tendency to form clusters that may settle at the tip of the needle.^50^ Therefore, it is essential to maintain an even cell suspension using agitation or other approaches.

*Administration protocols*. As cell injections for neurological injuries move on to extensive clinical testing, procedural standardisation is necessary to reduce the probability of technical failures and measure the effects of interventions on patient outcome and other endpoints (e.g. injection-associated trauma, graft survival). Clinical trial design to enhance the final number of cells that integrate within the diseased tissue will require re-examining the current lack of optimisation of transplantation protocols. For a given clinical trial, optimum factors for cell implantation are normally estimated from pre-clinical research or the investigator’s judgement.^80^ Administration protocols will vary depending on cell type, disease and administration route. While inappropriately administered cells may in most cases result in no functional impact, in some cases, transplanted cells may result in undesirable side effects.

*Injection rate* Injection rates employed in clinical trials for cell injection are inconsistent. For neural cell transplantation, for instance, studies have used a rate of 5 µL/min,^81^ compared to 300 µL/min for spinal injury,^82^ and between 10 and 1000 µL/min for stroke.^7, 83, 84^ Rate of injection has been shown to be potentially vital in cellular delivery.^50, 60, 74^

Although small volume injections may be made over extended periods in the laboratory, clinical injections must be made within limited surgical duration. It has been suggested that delivering the required volume over a longer time will potentially reduce mechanical forces acting on the cells and the creation of damaging pressure gradients^21^; delivery of 44 nL in 5 min was linked to good tissue preservation.^85^ However, such a rate would be impractical, with 1 μL requiring over an hour to deliver. Notably, brain microinjections in rats at a rate >1 μL/min have been linked to tissue damage.^86^ Recently, intracarotid transplantation of glial-restricted precursors (GRPs) and MSCs through a microcatheter at an infusion rate of ≥1 mL/min resulted in stroke in 27/44 rats, even with a vehicle-only infusion. A lower rate (0.2 mL/min) was safe for the infusion of both vehicle and GRPs.^87^ Moreover, at high flow rates, backflow along the catheter shaft may occur if the applied force is removed from the plunger and affect delivery. It was determined that backflow can be avoided in grey matter with a 32 G catheter at rates <0.5 µL/min in a rat model.^88^ However, injection at a slow rate may lead to a lower percentage of the cell dose being delivered, higher apoptosis levels and influence the immunophenotype of delivered cells.^60, 74^

*Cells employed* Optimal injection parameters will vary depending on the morphological, physiological and growth characteristics of each cell type. Thus, it is not surprising that preparation and ejection protocols optimised for one type of cells are not necessarily applicable to others.^32, 60, 74^ When studying injection rates, smaller NIH-3T3 cells had an optimum ejection rate of 150 µL/min, whereby the maximum percentage of viable cell dose was delivered,^60^ whereas the relatively larger BMSCs had an optimal ejection rate of 300 µL/min.^74^ Some cell types, such as MSCs, may be especially disposed to clumping, leading to blockage of flow within the needle,^49^ whereas other cell types, e.g. NSCs, are sensitive to manipulation and undergo apoptosis easily. During handling, MSCs were observed to have a greater inclination than NPCs to come out of suspension and form tight clusters.^49^

*Tissue compliance* If injections are made into cavities, larger volumes can be introduced without generating large pressure gradients if fluid drainage from the cavity is adequate, i.e. match injection to lesion volumes.^89, 90^ Hydrodynamic injury will occur if intraparenchymal pressure surpasses the tensile limits of the parenchyma. Given that many microsyringes are manufactured to tolerate 1000 psi or more before failure, the likelihood of producing large tissue pressures during injection exists. Larger injection volumes also worsen the reflux of infused materials along the penetration tract,^91^ making cell dosing unpredictable in terms of numbers and final location, and contributing to injection-related injury.^21^ The low elastic modulus of brain tissue also provides little resistance to reflux of infusions.^34^

### Post-delivery complications: functional performance of injected cells

Given the number of clinical trials that use needle-based systems or catheter-based systems for cell delivery, surprisingly few studies have focused on the impact of small-bore needle injection on cell function. Transplantation studies have focused on the outcome of the trial rather than the variables that affected results. Investigating viability and potency of transplanted cells at the time of delivery is crucial, as a small level of cell death within a concentrated cell population could have a significant effect on the remaining viable portion through the release of cytotoxic agents.^92^ To date, research has been carried out to evaluate the impact of injection on cell functionality.^25, 29, 50, 52, 93–96^ Studies have highlighted parameters needed to achieve an adequate cell density for therapy, such as the period between cell preparation and implantation,^97^ injection rate, needle length and bore size^50^ and cell concentration.^70, 98^

There has been wide variability in how the effects of delivery devices on cell performance are assessed. Therefore, it is vital to develop standardised assays to consistently characterise cellular therapy products. Discrepancies in the employed delivery devices and administration methodologies has complicated comparisons and led to contradictory results. This is illustrated in Table 1, with some studies demonstrating that cell manipulation through a needle did not significantly affect viability,^25, 93, 95^ whereas other studies show that it did exert a significant effect.^94^ In one study, ejection of NIH-3T3 cells at 150 µL/min exhibited the optimal percentage of dose delivered,^60^ whereas another study on hMSCs^74^ showed an optimal recovery at the highest flow rate under investigation (300 µL/min). A study by Heng et al.^29^ showed reduced cell recovery at the higher flow rate (1600 µL/min), with large variation in their samples.

**Table 1:** Overview of investigations carried out to assess the effects of various injection parameters on cell viability and functionality

Aim of study	Experimental design	Cell type	Syringe and needle	Flow rate(s) and other parameters	Brief description of results	Assays for assessment of cellular health				Refs.
						Viability	Apoptosis	Senescence	Others	
Viability after cell transfusion: various needles and flow rates	In vitro	Bone marrow-derived mono-nuclear cells	Automatic injection pump and 16, 18, 22 G needles	1 and 0.5 mm/s	No difference detected in viability ratios	√	×	×	×	95
Changes in hMSCs by transcatheter injection	In vitro	hMSCs	1-mL syringe and 26 G (155 cm) Nitinol needle	400 or 1600 µL/min	Viability not affected by varying rate. Slightly altered gene expression, but effects not translated into significant differences in protein production	√	×	×	Clonogenicity, gene expression profile and cytokine secretion	29
Response after manipulation in narrow-bore syringe system	In vitro	Murine MSCs	10 μL syringes and 26, 25 or 22 G needles	Drawn up at 30 μL/min; ejected at 20, 5 and 1 μL/min; time within syringe	Needle bore size and time within the syringe affected viable cell density	√	√	×	Cell attachment and spreading	94
Effect of delivery via needles and catheters at multiple flow rates	In vitro	Rat and human MSCs	20, 25 and 30 G needles, and SL-10 microcatheter	60, 120, 240 and 500 mL/h	No significant effect on viability (>70%). Delayed drop in viability at 24 h. No change in cell surface markers or function	√	√	×	Immunophenotyping and multilineage differentiation	25
Small-bore size to deliver single/multiple cell injections	In vitro and in vivo (IV by tail vein injection)	hMSCs	24, 25 and 26 G needles and 1 mL syringe. Multiple injections (10×): 26 G needle and 1 mL syringe	2000 μL/min	26 G needles can be safely used. Multiple injections were non-detrimental to cells (kept functional characteristics)	√	×	√	Morphology, immunophenotyping, trilineage differentiation, in vivo migration	93
Impact of manual handling procedures	In vitro	Mouse ESC cell lines	20 mL syringes: one containing cell suspension, luer-locked to stainless steel capillary (500 µm *D*, 1 cm *L*)	Pass cells between syringes at 0.80 mL/s. Centrifugation: 300×*g*, 600×*g* and 1000×*g*.Inoculation cell density	Gentle cell handling and minimal variations in environmental conditions needed to maintain viability. Inoculation density and time exposed to ambient conditions impacted phenotype.	√	×	×	Phenotyping	73
Impact of injection parameters in automated delivery for the brain	In vitro	Neural progenitor cells and bone marrow stromal cells	Automated injection device; 250 µL syringes with 20 G and 27 G needles, 3.8 or 15.2 cm in length	Flow rate of 1 µL or 10 µL/s; initial acceleration rate of 42 or 208 µL/s^2^.Delay between loading and injection	Longer, thinner cannulas and greater cell concentrations were harmful for delivery	√	×	×	×	50
Effect of DMSO, cell density and needle size on viability in 3D hydrogels	In vitro	NIH-3T3 cells	27 G needle	—	Viability of cryopreserved cells was significantly lower than freshly collected cells. Needle significantly reduced cell survival rates. Higher DMSO concentration and cell density lowered survival	√	×	×	×	96
Effect of varying ejection rate, cell density and needle gauge on cell health	In vitro	NIH-3T3 cells	30 and 34 G needles attached to 100 µL syringes	Drawn up at 300 µL/min, and ejected at 20–300 µL/min controlled using a syringe pump	Ejections at 150 μL/min resulted in highest percentage of dose delivered. Difference in proportions of apoptotic cells 48 h post-ejection was higher at slower rates	√	√	√	Cytotoxicity	60
Investigation of cell suspensions in large injection cannulas oriented at various angles	In vitro	Primary rat embryonic cell suspensions of neural tissue	18, 21 and 25 G metal cannulas. Glass cannulaswith nominal ID of 0.8 mm. Cannulas attached via a short siliconetubing to a 100-µL Hamilton syringe	10 µL/min using a syringe pump. Delay of 20 mins between loading and injection	Cell behaviour was affected by cannula diameter, orientation (horizontal/vertical) and material	×	×	×	Mean cell counts	75
Effect of transcatheter injections on cell viability and cytokine release	In vitro	Mononuclear cells	Cell suspension was aspirated into a 5-mL syringe and then infused through a 25 G needle. Cells were passed through an Excelsior SL-10 catheter; Iodine and heparin exposure	0.5–5 mL/min	Flow rates from 0.5–2 mL/min did not alter viability, but 5 mL/min reduced viability by 19%. Catheter delivery at 2 mL/min did not affect VEGF, IL-10 or IGF-1 levels. Iodine and low-dose heparin did not affect viability, but high-dose heparin caused cell death	√	√	×	Cellular function was assessed by production and release of VEGF, IL-10 and IGF-1	118
Effect of ejection rate and needle gauge on cell health	In vitro	hMSCs	30 and 34 G needles attached to 100 µL syringes	Drawn up at 300 µL/min, and ejected at 10–300 µL/min using a syringe pump	300 μL/min resulted in highest viable cell recovery. Apoptosis levels at 10 µL/min were significantly higher than control. Downregulation of CD105 expression at 10 µL/min	√	√	√	Immunophenotyping, trilineage differentiation	74

*CBF* cerebral blood flow, *G* gauge, *IV* intravenous.

Another potential concern is the inadequate testing of many aspects of cellular health in most studies, thereby not providing the complete picture needed to develop appropriate clinical administration protocols. Table 1 provides a summary of the investigations carried out into the effects of injection-based administration of cells on various parameters of cell health. Conventional tests, such as propidium iodide, can reveal which cells are dead, but are not useful predictors of potential delayed damage to the cells. The transient exposure to shear forces when injected does not make membrane integrity measurements, such as trypan blue exclusion assay, a good method of uncovering delayed apoptotic and senescent responses triggered. This is evident in some studies that have only relied on the analysis of cell viability to conclude that cells were not affected by the injection parameters under investigation, as shown in Table 1.^50, 95, 96^ In addition, some studies have utilised a relatively small sample size of *n* ≤ 3 for their investigations.^25, 29, 94^ Moreover, different studies had different definitions of what constitutes effective cell transplantation. Although the Centre for Biologics Evaluation and Research (CBER) states that cellular therapy products should display ≥70% viability,^99^ a study by Kondziolka et al.,^45^ considered a reduction of almost 50% in viability of cells post-injection acceptable.

The aforementioned issues illustrate that an enhanced understanding of what happens to cellular therapeutics post-injection, specifically regarding vital cellular health parameters, will facilitate the development of more efficient administration and formulation approaches. This reinforces the need for defining crucial parameters and trial-specific pre-clinical good laboratory practice validation of any injection protocol before human application. One method to enhance cell functionality testing in pre-clinical and clinical studies is to assess the viability of a small aliquot of injected cells. Further investigations into proliferative capacity, phenotypic expressions, apoptotic responses and other transcription-level changes of the various cell types under clinical investigation is critical. Without this, the uncertainty of whether delivery was effective will undermine interpretations of efficacy.

Most investigations carried out on the impact of injection on cell functionality and viability are completely in vitro, and although these data are important, other issues can undermine cell viability in vivo. For example, reflux and rejection can eliminate grafts in animals. In vitro experiments presented in this review will need to be augmented with in vivo data. We cannot exclude the possibility that cells may experience significant biological changes on catheter/syringe/needle passage outside the conditions examined in studies cited in this review. Optimisation of injection protocols, materials from which delivery devices are constructed, injection rates and cell dose is therefore crucial to achieve higher efficacy and reduce variability using the smallest possible cell dose.

## Approaches to improve injectability of cells

### Improvement of neurological cell transplantation protocols and tools

To decrease variations encountered in manual injection, automated devices (e.g. computer-controlled syringe pumps) have been suggested to offer better control.^49, 100^ Automated brain injections were more reproducible compared to manual injections, with variability 2–20-fold higher in manual techniques.^49^ For cell aggregates or encapsulated cells, automated cell-delivery methods may prove useful to control the cell dose and preserve structure integrity. However, manual delivery is still often preferred to automated robot-assisted tools due to the lack of haptic feedback to the surgeon in the latter method.^101^

To overcome the need for multiple injections, Mendez et al.^48^ developed a two-hole cannula tip design, whereas Lim and colleagues created a system capable of radially branched deployment (RBD) of a catheter at adjustable angles.^22, 34^ Notably, cells at high density (6 × 10^7^ cells/mL) were not damaged by transit at a high rate of delivery (50 µL/min, 99.6% viability).^34^ Although it represents an improvement, RBD still does not fully repeat the cell distribution achieved in pre-clinical models.

The use of syringe/cannula rotation during the injection procedure is a strategy described in the literature.^45, 75, 102^ In a clinical trial for Parkinson’s disease, the injection protocol incorporated rotations of the cannula between deposits, with transplant survival confirmed up to 12 months after surgery.^48^ Skewed distribution of cells in horizontally oriented cannulas can also be amended by rotation at regular intervals during the procedure.^75^ Robot-assisted surgery, image-based needle guidance systems can also facilitate accurate delivery. These include needles that automatically stop and lock into position by sensing drops in mechanical resistance.^103^ Technological improvements to platform and cannula design have reduced procedural invasiveness while improving injection accuracy, resulting in progress from single unilateral microinjections to multiple bilateral injections without long-term neurological consequences.^51^

Few clinical trials thoroughly optimise and state their cell transplantation protocols, and do not specify infusion volumes, rates or duration of administration, which may lead to variability and lower transplantation efficiency (Table 2). However, recent studies are beginning to recognise the importance of optimisation of transplantation protocols, both in pre-clinical and clinical trials.^87, 98, 104–107^ In a model of retinal dysfunction, photoreceptor integration was 20-fold to 30-fold increased by improved transplantation procedures including single and dual injections, and optimisation of the number of cells injected per µL.^108^ Another study defined optimised conditions for an autologous stem cell therapy to treat a craniofacial traumatic deficiency, regenerating 80% of the original jawbone deficiency in only 4 months vs. a minimum healing period of 6–8 months with typical protocols.^104^

**Table 2:** Selection of clinical trials for neural applications carried out using injectable cell therapy

Cells	Application	Route of administration	Injection device	Cell dose	Volume injected	Flow rate	Outcome	Refs.
MSCs	Amyotrophic lateral sclerosis	Intraspinal	Syringe with 18 G cannula needle mounted on a table fixed arm with a micrometric system. Cannula pre-modified to inject upwards and downwards	110 × 10^6^ cells. During treatment, different cell numbers were obtained in each subject. Only one patient received <15 × 10^6^ cells	Cells suspended in about 1 mL of autologous CSF	Not stated	MSC transplantation into the spinal cord is safe, but no definitive conclusion about cell vitality after transplantation	119
	Parkinson’s disease	Direct transplantation into the midbrain	Each patient was mounted with a Leksell stereotactic headframe. A 50 μL Hamilton syringe, fitted with a custom-made microinjector. Cell suspension was deposited along each of four putaminal trajectories	Final cell concentration of ≈80,000/μL. Total of 3.2 × 10^6^ cells in one patient and about 4.8 × 10^6^ cells in the other	E.g.: In the first patient, 40 μL injected along four tracks in the right postcommissural putamen, and 32 μL in the left	Not stated	Results demonstrate that such therapies can be effective in some patients at advanced stages of disease. Changes in methodology may result in better clinical outcome	46
	Chronic spinal cord injury	Intraarterial	Cobra 2 catheter (tubular, polyurethane 4 Fr and 65 cm long)	2.5 × 10^6^ CD 34+ cells/kg	Not stated	10 mL/min	Recovery of somatosensory evoked response to peripheral stimuli in 67% of patients. During a 2.5-year follow-up, this protocol proved safe	120
		Intrathecal	Not stated	5 × 10^6^ to 10 × 10^6^/kg of mononuclear cells	Not stated	Not stated	No statistical improvement demonstrated. One case of encephalomyelitis after 3rd injection. 24 patients developed neuropathic pain	27
LBS-neurons	Ischaemic or haemorrhagic stroke	Intracerebral	0.9 mm-OD cannula with 20 µL. Cells were aspirated into 100 µL syringe	5 × 10^6^ or 1 × 10^7^ cells	10 µL was injected slowly at each site over 2 mins	5 µL/min. Total time was around 150 min	A quantifiable improvement was noted in some patients but no evidence of significant value in motor function	4
MSCs and NSPCs	Ischaemic stroke	Either four IV injections of MSCs or one IV injection of MSCs followed by three injections of MSCs and NSPCs through the cerebellomedullary cistern	Not described	Either four IV injections of MSCs at 0.5 × 10^6^/kg body weight; or one IV injection of MSCs at 0.5 × 10^6^/kg followed by three injections at 5 × 10^6^/patient and NSPCs at 6 × 10^6^/patient	IV injections of MSCs in 250 mL saline; and the injections of MSCs and NSPCs in 10 mL saline	Not stated	No evidence of neurological deterioration, Infection or tumorigenesis at a 2-year follow-up. Neurological functions and disability levels were improved	39
NSI-566RSC (Neuralstem, Inc)	Amyotrophic lateral sclerosis	Intraspinal	Microinjection platform base attached to a custom self-retaining retractor system. Five sequential unilateral injections	1 × 10^4^ cells/mL	5 injections of 10 µL at 4-mm intervals	Not stated	Delivery was well tolerated	121
Olfactory ensheathing cells	Complete, thoracic paraplegia	Intraspinal	25 µL Hamilton syringe with 28 G bevelled needle	80,000 cells/µL	Four injections of 1.1 µL during each penetration	Injections frame-assisted and freehand	Transplantations were feasible and safe up to 3 years post-implantation	43, 122
	Chronic thoracic paraplegia	Intraspinal	Automatic micropump and 3D micromanipulator, with 25 μL glass syringe and 26 G bevelled needle	30,000–200,000 cells/μL	Volume of single injections was 0.5 μL	2 μL/min	Neurological improvements in the three patients, with confirmation of significance requiring larger sample	42

This table is illustrative of the numerous clinical cell-therapy trials undertaken in the field of neurodegenerative diseases. Trials shown were selected to exemplify the range of therapies currently under investigation, and should not be taken as an indication of the quality of any particular trial.

*IV Intravenous*.

### Biomaterial-assisted delivery

Although many transplantation studies have employed a saline-based delivery vehicle, alternative cell carriers have included injectable hydrogels. Hydrogels are hydrated, polymeric networks with great potential as cell carriers.^109, 110^ Alternatives to saline vehicles have included cells embedded within hydrogels or microencapsulated within polymers, attached to the surfaces of microcarriers, or injected as multicellular aggregates.^111^ Injectable biomaterial scaffolds as cell carriers have demonstrated increased spatial and temporal administration compared to saline injections.^112, 113^ In addition, the use of biomaterials provides an opportunity to deliver growth factors alongside cells.

Hydrogels such as alginate may experience ‘shear banding’ along inner walls of the needle,^52^ whereby a layer of hydrogel shear-thins to form a fluid, acting as a lubricant, allowing the rest of the intact hydrogel to slip through the needle. The width of the plug flow region is dependent upon rigidity of the hydrogel and flow rate,^114^ therefore altering the hydrogel’s formulation may impact plug flow. This mechanical protection is independent of cell properties^52^ and is therefore applicable to different cell types. Aguado et al.^52^ tested 1% alginate with three different molecular weights, and demonstrated protective effects with optimised mechanical properties; human umbilical vein endothelial cells had significantly lower cell viability in phosphate-buffered saline or in non-crosslinked alginate compared to in crosslinked alginates.

Considerable research on hydrogel cell carriers has focused on their role post-delivery, including cell localisation,^110^ support of tissue growth^115, 116^ and protection from local inflammatory conditions.^117^ More focus is required on their role during the injection procedure, as opposed to post-delivery, where the presence of an optimised viscoelastic material may protect cells from damaging mechanical forces.^52, 60^ This has been only investigated on a small scale (Table 3), and needs to be explored further.

**Table 3:** Selection of investigations carried out into potential protective mechanisms for cell cargos

Aim of study	Cell type	Needle size	Flow rate	Brief description of results	Refs.
*Use of hydrogels*					
Improving viability during injection by alginate hydrogels	Human umbilical vein endothelial cells and adipose stem cells, rat MSCs, and mouse neural progenitor cells	28 G needle on 1 mL syringes	1000 µL/min	Crosslinked alginate hydrogel produced highest viability. Increasing or decreasing G′ reduced protective effect. Cells in non-crosslinked alginate exhibited lower viabilities than media. Data suggested extensional flow at needle entrance was chief cause of cell death	52
β-hairpin peptide hydrogel as carrier during syringe flow	MG63	26 G needle on 1 mL syringe	4, 6 and 8 mL/h	Only gel at the capillary wall experienced a velocity gradient, whereas the rest was subject to minimal shear rate. Hydrogels had no apparent effect on viability of encapsulated cells	114
Injectable fibrin matrix to enhance vascularisation	Bone marrow mononuclear cells (BMMNCs)	100 µL injection—Needle size not mentioned	Not mentioned	Device was constructed for simultaneous injection of fibrinogen and thrombin solutions. Implantation of BMMNCs in fibrin resulted in better tissue regeneration and neovascularisation	123
Growth factor supplemented matrigel for cell delivery	C2C12 myoblasts	Not mentioned	Slow-exact rate not mentioned	Results showed that the combination of matrigel as a cell carrier for myoblasts with growth factors is recommended for the generation of muscle in vivo	124
*Use of microparticles*					
PLGA particles for intracerebral delivery	Neural stem cells	22 G needle on a 50 µL gastight Luer-tip syringe	2 μL/min	Plasma polymerised allylamine-treated MPs were used. Cell attachment was influenced by curvature, material, electrostatic charge and surface motif of particles, and the number of cells in the culture	125, 126
Nerve growth factor (NGF)-releasing PLGA microparticles	Foetal rat (E16-E17) brain cells	22s-G needle on a 10 µL syringe	<1 µL/min	Dose of NGF delivered can be modified by changing quantity of microparticles or NGF release rate. Activity of neo-tissues with NGF-enriched microenvironments increased in in vivo and in vitro	127
*Automated vs. manual systems*					
Compare manual and automated injection	Neural progenitor cells and bone marrow stromal cells	Automated device for μL syringes (MEDRAD Inc.)	—	Automated delivery resulted in less variability in amount delivered. No significant difference in viability attributable to method of injection	49

*G′* hydrogel storage modulus.

## Concluding remarks and future outlook

The complexity of the cell injection process has resulted in a paucity of studies where combinations of process parameters have been evaluated. Nonetheless, these few studies demonstrate that interactions between cell preparation protocols and injection procedures are significant, and may substantially alter cell delivery. Studies also suggest that standardisation of injection parameters will be a critical aspect of designing and comparing clinical studies. In addition, factors such as choice of instrument, interval between filling of the cannula/needle and fixing to the stereotaxic frame prior to injection, and angles of delivery must be considered and empirically validated before use in clinical practice.

An integrated approach to the evaluation of cell-delivery success is needed to improve the assessment of delivery efficacy and to allow for sound interpretations of clinical results. Improved cell-delivery tools are also required to streamline the delivery of cell-based therapeutics from the donor to the patient without compromising quality. Finally, pre-clinical planning and testing of the desired administration protocol with cell-type specificity is essential to achieve good clinical trial design.

## References

[1] Olanow CW (2003). A double-blind controlled trial of bilateral fetal nigral transplantation in Parkinson’s disease. Ann. Neurol..

[2] Bachoud-Levi AC (2006). Effect of fetal neural transplants in patients with Huntington’s disease 6 years after surgery: a long-term follow-up study. Lancet Neurol..

[3] Lunn JS, Sakowski SA, Hur J, Feldman EL (2011). Stem cell technology for neurodegenerative diseases. Ann. Neurol..

[4] Kondziolka D (2005). Neurotransplantation for patients with subcortical motor stroke: a phase 2 randomized trial. J. Neurosurg..

[5] Bliss TM, Andres RH, Steinberg GK (2010). Optimizing the success of cell transplantation therapy for stroke. Neurobiol. Dis..

[6] Jones LA (2010). A phase 2 autologous cellular therapy trial in patients with acute, complete spinal cord injury: pragmatics, recruitment, and demographics. Spinal Cord.

[7] Steinberg GK (2016). Clinical outcomes of transplanted modified bone marrow-derived mesenchymal stem cells in stroke: a phase 1/2a study. Stroke..

[8] Kalladka D (2016). Human neural stem cells in patients with chronic ischaemic stroke (PISCES): a phase 1, first-in-man study. Lancet.

[9] Srijaya TC, Ramasamy TS, Kasim NH (2014). Advancing stem cell therapy from bench to bedside: lessons from drug therapies. J. Transl. Med..

[10] Rossetti T, Nicholls F, Modo M (2015). Intra-cerebral cell implantation: preparation and characterization of cell suspensions. Cell Transplant..

[11] Karimi-Abdolrezaee S, Eftekharpour E, Wang J, Morshead CM, Fehlings MG (2006). Delayed transplantation of adult neural precursor cells promotes remyelination and functional neurological recovery after spinal cord injury. J. Neurosci..

[12] Paul C, Samdani AF, Betz RR, Fischer I, Neuhuber B (2009). Grafting of human bone marrow stromal cells into spinal cord injury a comparison of delivery methods. Spine.

[13] Hicks AU (2009). Transplantation of human embryonic stem cell-derived neural precursor cells and enriched environment after cortical stroke in rats: cell survival and functional recovery. Eur. J. Neurosci..

[14] Hubschman JP, Reddy S, Schwartz SD (2009). Age-related macular degeneration: current treatments. Clin. Ophthalmol..

[15] Wang G (2010). Analysis of the indel at the ARMS2 3’UTR in age-related macular degeneration. Hum. Genet..

[16] Laflamme MA, Murry CE (2011). Heart regeneration. Nature.

[17] Freed CR (2001). Transplantation of embryonic dopamine neurons for severe Parkinson’s disease. N. Engl. J. Med..

[18] Lindvall O, Bjorklund A (2004). Cell therapy in Parkinson’s disease. NeuroRx..

[19] Selden NR, G. D., Huhn S. L., Koch T. K., Al-Uzri A., Steiner R. D. (eds) *American Association of Neurological Surgeons Annual Meeting* (AANS, 2010).

[20] Silva EA, Kim ES, Kong HJ, Mooney DJ (2008). Material-based deployment enhances efficacy of endothelial progenitor cells. Proc. Natl Acad. Sci. USA.

[21] Guest J, Benavides F, Padgett K, Mendez E, Tovar D (2011). Technical aspects of spinal cord injections for cell transplantation. Clinical and translational considerations. Brain Res. Bull..

[22] Potts MB, Silvestrini MT, Lim DA (2013). Devices for cell transplantation into the central nervous system: design considerations and emerging technologies. Surg. Neurol. Int..

[23] Schwartz SD, Anglade E, Lanza R, Ocata Macular Disease Investigator Group. (2015). Stem cells in age-related macular degeneration and Stargardt’s macular dystrophy—authors’ reply. Lancet.

[24] Borlongan CV, Weiss MD (2011). Baby STEPS: a giant leap for cell therapy in neonatal brain injury. Pediatr. Res..

[25] Walker PA (2010). Effect of needle diameter and flow rate on rat and human mesenchymal stromal cell characterization and viability. Tissue Eng. Part. C. Methods.

[26] Yamout B (2010). Bone marrow mesenchymal stem cell transplantation in patients with multiple sclerosis: a pilot study. J. Neuroimmunol..

[27] Kishk NA (2010). Case control series of intrathecal autologous bone marrow mesenchymal stem cell therapy for chronic spinal cord injury. Neurorehabil. Neural Repair.

[28] Taguchi A (2015). Intravenous autologous bone marrow mononuclear cell transplantation for stroke: phase1/2a clinical trial in a homogeneous group of stroke patients. Stem Cells Dev..

[29] Heng BC (2009). Transcatheter injection-induced changes in human bone marrow-derived mesenchymal stem cells. Cell Transplant..

[30] Shehadah A (2014). Human placenta-derived adherent cell treatment of experimental stroke promotes functional recovery after stroke in young adult and older rats. PLoS ONE.

[31] Chen Y (2013). Effects of storage solutions on the viability of human umbilical cord mesenchymal stem cells for transplantation. Cell Transplant..

[32] Watts C, Caldwell MA, Dunnett SB (1998). The development of intracerebral cell-suspension implants is influenced by the grafting medium. Cell Transplant..

[33] Mathew AJ, Baust JM, Van Buskirk RG, Baust JG (2004). Cell preservation in reparative and regenerative medicine: evolution of individualized solution composition. Tissue Eng..

[34] Silvestrini MT (2013). Radially branched deployment for more efficient cell transplantation at the scale of the human brain. Stereotact. Funct. Neurosurg..

[35] Breeze RE, Wells TH, Freed CR (1995). Implantation of fetal tissue for the management of Parkinson’s disease: a technical note. Neurosurgery.

[36] Miyanji F, Furlan JC, Aarabi B, Arnold PM, Fehlings MG (2007). Acute cervical traumatic spinal cord injury: MR imaging findings correlated with neurologic outcome—prospective study with 100 consecutive patients. Radiology.

[37] Gutierrez J (2015). Preclinical validation of multilevel intraparenchymal stem cell therapy in the porcine spinal cord. Neurosurgery.

[38] Usvald D (2010). Analysis of dosing regimen and reproducibility of intraspinal grafting of human spinal stem cells in immunosuppressed minipigs. Cell Transplant..

[39] Qiao LY (2014). A two-year follow-up study of cotransplantation with neural stem/progenitor cells and mesenchymal stromal cells in ischemic stroke patients. Cell Transplant..

[40] Miljan EA, Sinden JD (2009). Stem cell treatment of ischemic brain injury. Curr. Opin. Mol. Ther..

[41] Lammertse DP (2012). Autologous incubated macrophage therapy in acute, complete spinal cord injury: results of the phase 2 randomized controlled multicenter trial. Spinal Cord.

[42] Tabakow P (2013). Transplantation of autologous olfactory ensheathing cells in complete human spinal cord injury. Cell Transplant..

[43] Feron F (2005). Autologous olfactory ensheathing cell transplantation in human spinal cord injury. Brain.

[44] Knoller N (2005). Clinical experience using incubated autologous macrophages as a treatment for complete spinal cord injury: phase I study results. J. Neurosurg-Spine.

[45] Kondziolka D, Steinberg GK, Cullen SB, McGrogan M (2004). Evaluation of surgical techniques for neuronal cell transplantation used in patients with stroke. Cell Transplant..

[46] Mendez I (2005). Cell type analysis of functional fetal dopamine cell suspension transplants in the striatum and substantia nigra of patients with Parkinson’s disease. Brain.

[47] Bjarkam CR (2010). Safety and function of a new clinical intracerebral microinjection instrument for stem cells and therapeutics examined in the Gottingen minipig. Stereotact. Funct. Neurosurg..

[48] Mendez I, Hong M, Smith S, Dagher A, Desrosiers J (2000). Neural transplantation cannula and microinjector system: experimental and clinical experience. J. Neurosurg..

[49] Gobbel GT, Kondziolka D, Fellows-Mayle W, Uram M (2010). Manual vs automated delivery of cells for transplantation: accuracy, reproducibility, and impact on viability. Neurosurgery.

[50] Kondziolka D, Gobbel GT, Fellows-Mayle W, Chang YF, Uram M (2011). Injection parameters affect cell viability and implant volumes in automated cell delivery for the brain. Cell Transplant..

[51] Riley JP, Raore B, Taub JS, Federici T, Boulis NM (2011). Platform and cannula design improvements for spinal cord therapeutics delivery. Neurosurgery.

[52] Aguado BA, Mulyasasmita W, Su J, Lampe KJ, Heilshorn SC (2012). Improving viability of stem cells during syringe needle flow through the design of hydrogel cell carriers. Tissue Eng. Part. A.

[53] Grellier M, Bareille R, Bourget C, Amedee J (2009). Responsiveness of human bone marrow stromal cells to shear stress. J. Tissue Eng. Regen. Med..

[54] Stolberg S, McCloskey KE (2009). Can shear stress direct stem cell fate?. Biotechnol. Prog..

[55] Bird, R. B., Stewart, W. E. & Lightfoot, E. N. *Transport Phenomena* (Wiley, 2007).

[56] Reneman RS, Hoeks AP (2008). Wall shear stress as measured in vivo: consequences for the design of the arterial system. Med. Biol. Eng. Comput..

[57] Malek AM, Alper SL, Izumo S (1999). Hemodynamic shear stress and its role in atherosclerosis. JAMA.

[58] Korenaga R (1997). Negative transcriptional regulation of the VCAM-1 gene by fluid shear stress in murine endothelial cells. Am. J. Physiol..

[59] Wang H (2005). Shear stress induces endothelial differentiation from a murine embryonic mesenchymal progenitor cell line. Arterioscler. Thromb. Vasc. Biol..

[60] Amer MH, White LJ, Shakesheff KM (2015). The effect of injection using narrow-bore needles on mammalian cells: administration and formulation considerations for cell therapies. J. Pharm. Pharmacol..

[61] Doran, P. M. in *Bioprocess Engineering Principles* 2nd edn. 1–919 (Elsevier, 2013).

[62] Sutera SP (1977). Flow-induced trauma to blood cells. Circ. Res..

[63] Lee SS, Yim Y, Ahn KH, Lee SJ (2009). Extensional flow-based assessment of red blood cell deformability using hyperbolic converging microchannel. Biomed. Microdevices.

[64] Tanzeglock T, Soos M, Stephanopoulos G, Morbidelli M (2009). Induction of mammalian cell death by simple shear and extensional flows. Biotechnol. Bioeng..

[65] Muller-Ehmsen J (2002). Survival and development of neonatal rat cardiomyocytes transplanted into adult myocardium. J. Mol. Cell Cardiol..

[66] Mazzini L (2015). Human neural stem cell transplantation in ALS: initial results from a phase I trial. J. Transl. Med..

[67] Riley J (2012). Intraspinal stem cell transplantation in amyotrophic lateral sclerosis: a phase I safety trial, technical note, and lumbar safety outcomes. Neurosurgery.

[68] Ballios BG, Cooke MJ, van der Kooy D, Shoichet MS (2010). A hydrogel-based stem cell delivery system to treat retinal degenerative diseases. Biomaterials.

[69] van Asten F (2015). Are intravitreal injections with ultrathin 33-G needles less painful than the commonly used 30-G needles?. Retina.

[70] Nikkhah G (1994). A microtransplantation approach for cell suspension grafting in the rat Parkinson model: a detailed account of the methodology. Neuroscience.

[71] Mehta T (2008). Subarachnoid placement of stem cells in neurological disorders. Transplant. Proc..

[72] Karussis D (2010). Safety and immunological effects of mesenchymal stem cell transplantation in patients with multiple sclerosis and amyotrophic lateral sclerosis. Arch. Neurol..

[73] Veraitch FS, Scott R, Wong JW, Lye GJ, Mason C (2008). The impact of manual processing on the expansion and directed differentiation of embryonic stem cells. Biotechnol. Bioeng..

[74] Amer MH, Rose FR, White LJ, Shakesheff KM (2016). A detailed assessment of varying ejection rate on delivery efficiency of mesenchymal stem cells using narrow-bore needles. Stem Cells Transl. Med..

[75] Torres EM, Trigano M, Dunnett SB (2015). Translation of cell therapies to the clinic: characteristics of cell suspensions in large-diameter injection cannulae. Cell Transplant..

[76] Heaton JT (2012). Modification and testing of a pneumatic dispensing device for controlled delivery of injectable materials. Laryngoscope.

[77] Guo BL, Finne-Wistrand A, Albertsson AC (2010). Molecular architecture of electroactive and biodegradable copolymers composed of polylactide and carboxyl-capped aniline trimer. Biomacromolecules.

[78] Bayoussef Z, Dixon JE, Stolnik S, Shakesheff KM (2012). Aggregation promotes cell viability, proliferation, and differentiation in an in vitro model of injection cell therapy. J. Tissue Eng. Regen. Med..

[79] Ma Y (2002). Dyskinesia after fetal cell transplantation for parkinsonism: a PET study. Ann. Neurol..

[80] Borlongan CV (2008). Potential of stem/progenitor cells in treating stroke: the missing steps in translating cell therapy from laboratory to clinic. Regen. Med..

[81] Glass JD (2012). Lumbar intraspinal injection of neural stem cells in patients with amyotrophic lateral sclerosis: results of a phase I trial in 12 patients. Stem Cells.

[82] Yoon SH (2007). Complete spinal cord injury treatment using autologous bone marrow cell transplantation and bone marrow stimulation with granulocyte macrophage-colony stimulating factor: phase I/II clinical trial. Stem Cells.

[83] Misra V, Lal A, El Khoury R, Chen PR, Savitz SI (2012). Intra-arterial delivery of cell therapies for stroke. Stem Cells Dev..

[84] Rosado-de-Castro PH, Pimentel-Coelho PM, da Fonseca LM, de Freitas GR, Mendez-Otero R (2013). The rise of cell therapy trials for stroke: review of published and registered studies. Stem Cells Dev..

[85] Myers RD (1966). Injection of solutions into cerebral tissue - relation between volume and diffusion. Physiol. Behav..

[86] Peterson SL (1998). Drug microinjection in discrete brain regions. Kopf Carrier.

[87] Janowski M (2013). Cell size and velocity of injection are major determinants of the safety of intracarotid stem cell transplantation. J. Cerebr. Blood Flow Metab..

[88] Morrison PF, Chen MY, Chadwick RS, Lonser RR, Oldfield EH (1999). Focal delivery during direct infusion to brain: role of flow rate, catheter diameter, and tissue mechanics. Am. J. Physiol..

[89] Massensini AR (2015). Concentration-dependent rheological properties of ECM hydrogel for intracerebral delivery to a stroke cavity. Acta Biomater..

[90] Nicholls FJ, Ling W, Ferrauto G, Aime S, Modo M (2015). Simultaneous MR imaging for tissue engineering in a rat model of stroke. Sci. Rep..

[91] Yin D (2011). Optimal region of the putamen for image-guided convection-enhanced delivery of therapeutics in human and non-human primates. Neuroimage.

[92] Agashi, K. *The Analysis of Cell Fate Post-Ejection through Parenteral Devices and the Development of Systems that Aid the Transportation of Cell Therapy Products*. PhD thesis, University of Nottingham (2010).

[93] Mamidi MK (2012). Impact of passing mesenchymal stem cells through smaller bore size needles for subsequent use in patients for clinical or cosmetic indications. J. Transl. Med..

[94] Agashi K, Chau DYS, Shakesheff KM (2009). The effect of delivery via narrow-bore needles on mesenchymal cells. Regen. Med..

[95] Tol M, Akar AR, Durdu S, Ayyildiz E, Ilhan O (2008). Comparison of different needle diameters and flow rates on bone marrow mononuclear stem cell viability: an ex vivo experimental study. Cytotherapy.

[96] Chen X, Thibeault S (2013). Effect of DMSO concentration, cell density and needle gauge on the viability of cryopreserved cells in three dimensional hyaluronan hydrogel. Conf. Proc. IEEE Eng. Med. Biol. Soc..

[97] Gobbel GT, Kondziolka D, Fellows-Mayle W, Uram M (2010). Cellular transplantation for the nervous system: impact of time after preparation on cell viability and survival. J. Neurosurg..

[98] Nikkhah G (2009). Microtransplantation of dopaminergic cell suspensions: further characterization and optimization of grafting parameters. Cell Transplant..

[99] Center for Biologics Evaluation and Research. *Guidance for FDA Reviewers and Sponsors: Content and Review of Chemistry, Manufacturing, and Control (CMC) Information for Human Gene Therapy Investigational New Drug Applications (INDs)* (Food and Drug Administration, US Department of Health and Human Services, 2008).

[100] Richard PL (2010). A first semimanual device for clinical intramuscular repetitive cell injections. Cell Transplant..

[101] Okamura AM (2009). Haptic feedback in robot-assisted minimally invasive surgery. Curr. Opin. Urol..

[102] Brundin P (2000). Bilateral caudate and putamen grafts of embryonic mesencephalic tissue treated with lazaroids in Parkinson’s disease. Brain.

[103] Schwartz SG (2014). Re: Awh *et al*.: CFH and ARMS2 Genetic polymorphisms predict response to antioxidants and zinc in patients with age-related macular degeneration (*Ophthalmology* 2013;**120**:2317–2323). Ophthalmology.

[104] Rajan A (2014). Optimized cell survival and seeding efficiency for craniofacial tissue engineering using clinical stem cell therapy. Stem Cells Transl. Med..

[105] Zakaria N (2014). Results of a phase I/II clinical trial: standardized, non-xenogenic, cultivated limbal stem cell transplantation. J. Transl. Med..

[106] Reyes S, Tajiri N, Borlongan CV (2015). Developments in intracerebral stem cell grafts. Expert Rev. Neurother..

[107] Harting MT, Sloan LE, Jimenez F, Baumgartner J, Cox CS (2009). Subacute neural stem cell therapy for traumatic brain injury. J. Surg. Res..

[108] Pearson RA (2012). Restoration of vision after transplantation of photoreceptors. Nature.

[109] Guarino V, Gloria A, Raucci MG, Ambrosio L (2012). Hydrogel-based platforms for the regeneration of osteochondral tissue and intervertebral disc. Polymers.

[110] Seif-Naraghi SB, Salvatore MA, Schup-Magoffin PJ, Hu DP, Christman KL (2010). Design and characterization of an injectable pericardial matrix gel: a potentially autologous scaffold for cardiac tissue engineering. Tissue Eng. Part A.

[111] Bidarra SJ, Barrias CC, Granja PL (2014). Injectable alginate hydrogels for cell delivery in tissue engineering. Acta Biomater..

[112] Slaughter BV, Khurshid SS, Fisher OZ, Khademhosseini A, Peppas NA (2009). Hydrogels in regenerative medicine. Adv. Mater..

[113] Tan HP, Marra KG (2010). Injectable, biodegradable hydrogels for tissue engineering applications. Materials.

[114] Yan C (2012). Injectable solid peptide hydrogel as a cell carrier: effects of shear flow on hydrogels and cell payload. Langmuir.

[115] Kong HJ, Smith MK, Mooney DJ (2003). Designing alginate hydrogels to maintain viability of immobilized cells. Biomaterials.

[116] Bible E (2012). Non-invasive imaging of transplanted human neural stem cells and ECM scaffold remodeling in the stroke-damaged rat brain by (19)F- and diffusion-MRI. Biomaterials.

[117] Zeng Q, Chen W (2010). The functional behavior of a macrophage/fibroblast co-culture model derived from normal and diabetic mice with a marine gelatin-oxidized alginate hydrogel. Biomaterials.

[118] El Khoury R (2010). The effect of transcatheter injections on cell viability and cytokine release of mononuclear cells. AJNR.

[119] Mazzini L (2010). Mesenchymal stem cell transplantation in amyotrophic lateral sclerosis: a phase I clinical trial. Exp. Neurol..

[120] Cristante AF (2009). Stem cells in the treatment of chronic spinal cord injury: evaluation of somatosensitive evoked potentials in 39 patients. Spinal Cord.

[121] Riley J (2014). Intraspinal stem cell transplantation in amyotrophic lateral sclerosis: a phase I trial, cervical microinjection, and final surgical safety outcomes. Neurosurgery.

[122] Mackay-Sim A (2008). Autologous olfactory ensheathing cell transplantation in human paraplegia: a 3-year clinical trial. Brain.

[123] Ryu JH (2005). Implantation of bone marrow mononuclear cells using injectable fibrin matrix enhances neovascularization in infarcted myocardium. Biomaterials.

[124] Barbero A (2001). Growth factor supplemented matrigel improves ectopic skeletal muscle formation--a cell therapy approach. J. Cell Physiol..

[125] Bible E (2009). Attachment of stem cells to scaffold particles for intra-cerebral transplantation. Nat. Protoc..

[126] Bible E (2009). The support of neural stem cells transplanted into stroke-induced brain cavities by PLGA particles. Biomaterials.

[127] Mahoney MJ, Saltzman WM (2001). Transplantation of brain cells assembled around a programmable synthetic microenvironment. Nat. Biotechnol..

